# Regenerative endodontic procedure combined with apical surgery of a necrotic permanent incisor with extensive periapical lesion using plasma rich in growth factors (PRGF): A Case report with 6 years post-op evaluation using CBCT

**DOI:** 10.4317/jced.58113

**Published:** 2021-06-01

**Authors:** José-Francisco Gaviño-Orduña, Inés Fernández-Guallart, Javier Caviedes-Bucheli, Manuel Espadas-García, José López-López

**Affiliations:** 1 DDs, PhD student. Dentist. Department of Odonto-Stomatology, School of Dentistry, University of Barcelona; 2 DDs, MsC Dentist. Department of Odonto-Stomatology, School of Dentistry, University of Barcelona; 3 DDs, MsC Centro de Investigaciones Odontologicas (CIO) Pontificia Universidad Javeriana Bogota Colombia; 4 MD, DDS, Dentist. Department of Odonto-Stomatology, School of Dentistry, University of Barcelona; 5 PhD, MD, DDS, Dentist. University of Barcelona. Spain. Professor of Oral Patholoy. School of Dentistry, Barcelona University / Oral Health and Masticatory System Group (Bellvitge Biomedical Research Institute) IDIBELL, University of Barcelona, L’Hospitalet de Llobregat, Barcelona 08907, Spain

## Abstract

**Background:**

The aim of this case report is to describe the step-by step and outcomes of a treatment approach which simultaneously combines a Regenerative Endodontic Procedure (REP) and apical surgery using PRGF as treatment of a post-traumatic necrotic permanent central incisor with extensive periapical lesion and undeveloped apex in a 16 years-old patient.

**Case description:**

A 16 years-old patient with an extensive periapical lesion in the maxillary central incisor was treated with a combination of REP and periapical surgery in the same visit. A Bi- antibiotic paste (BAP) was used to priorly disinfect the canal, which was posteriorly sealed with MTA placed over a PRGF clot at the same time that the periapical lesion was surgically debrided, removed and grafted, The radiographic and CBCT follow-up results showed a complete healing of the radiolucent lesion, which was replaced with bone-like tissue in two years and well preserved until six years later.

**Practical implications:**

A combined approach of a Regenerative Endodontic Procedure and apical surgery using PRGF may be a good treatment modality in cases of extensive periapical lesions in necrotic teeth with open apexes. PRGF has shown to act as an ideal autologous matrix because it is stable, provides growth factors and bioactive molecules, and stimulates collagen production, angiogenesis, and cell differentiation.

** Key words:**Regenerative endodontic procedure, open apex necrotic tooth, periodontal apical lesion, growth factors, apical surgery.

## Introduction

Early dental trauma to immature teeth can result in loss of neurovascular supply, leading to interruption of rhizogenesis and the consequent weakening of the root structure, which can lead to fracture in 77% of the reported treated cases (1).

The choice between nonsurgical treatment and endodontic surgery is based on the benefits (capability to heal apical periodontitis) and risks associated to either alternative. This procedure combined with nonsurgical retreatment shows 1%-25% higher success rates than when endodontic surgery is performed alone (2).

Regenerative Endodontic Procedures (REPs) of necrotic immature teeth with open apexes have demonstrated predicTable clinical results, since they provide three advantages over traditional methods of apexification in terms of root development: increasing root length, thickening root dentinal walls, and achievement of an apical closure (3). Disinfection of the root canal, obtaining a biological scaffold by achieving bleeding into the canal and sealing it with a biocompatible material seem to be key factors to provide the necessary environment for the success of REPs (3). In recent publications, Plasma Rich in Growth Factors (PRGF®-Endoret®) used as a scaffold has shown the capability to stimulate collagen production, angiogenesis, and cell differentiation as well as anti-inflammatory and anti-bacterial properties both in REP and surgical treatment (4).

The aim of this case report is to describe the step-by step and outcomes of a single stage approach combining REP and apical surgery using PRGF as treatment of a post-traumatic necrotic permanent central incisor with extensive periapical lesion and undeveloped apex in a 16 years-old patient.

## Case Report

**Figure 1:**
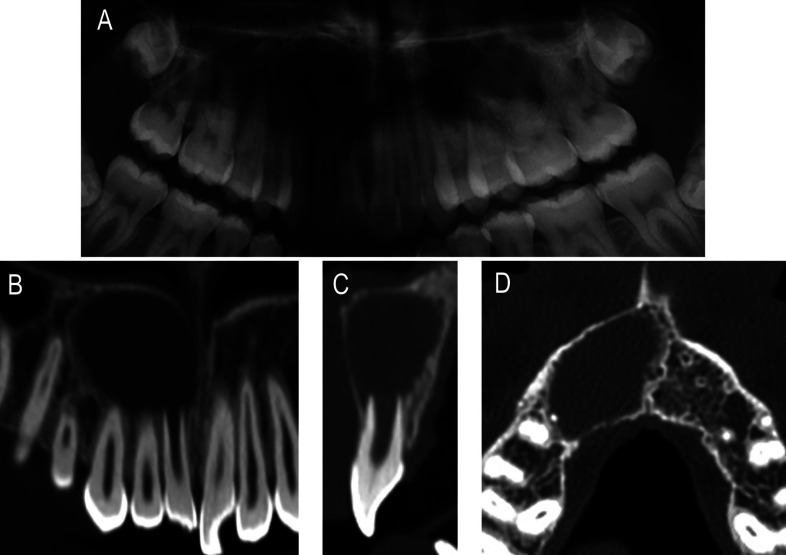
A) Pre-op orthopantomography ilustrating a extensive radiolucent lesion in the upper right maxilla related to the apices of 1.1, 1.2, and 1.3. (B-D) Selected images of computed tomography B) Coronal slice showing the lesion affecting from 1.1 to 1.5 C) Sagital slice show the lesion in relation with open apex 1.1 D) Axial slice show extensive lesion occupying bone marrow in the upper right maxilla and bulging cortical bone plates.

A 16-year-old male patient presented to the Department of Endodontics at the University of Barcelona referred by the Orthodontics Department. The patient had a non-remarkable medical history except for a traumatism occurred ten years before, in which he hit his front teeth. At the time, no dental treatment was performed, and since he did not have symptoms in the following years, he did not follow-up either. Upon examination during his orthodontics consultation, an asymptomatic extensive radiolucent lesion in the upper right maxilla was noticed in the panoramic X-ray (Fig. 1A).

The endodontic clinical examination revealed staining and an untreated enamel-dentin fracture on the maxillary right central incisor. The percussion test showed an augmented response for this tooth, while the cold vitality test (Endo Ice, Hygenic, Coltène/Whaledent Inc. Cuyahoga Falls, Ohio) was negative, being positive for the neighbouring teeth. No spontaneous pain was referred, and periodontal probing depths (CP-12, Hu-Friedy, Chicago, USA) were 2mm all around the tooth, with no signs of mobility. A periapical radiograph (PA) using a paralleling technique revealed an incomplete root development showing an open apex for the right central incisor, corresponding to Nolla’s eighth stage and associated with a large periapical radiolucency. The computed tomography evaluation (CBCT) confirmed the presence of an extensive periapical lesion (19 x 12 x 16 mm) and its intimate contact with the apexes of the maxillary right central and lateral incisors (Fig. 1B-D). A small fenestration was observed affecting the buccal bony wall of the maxillary right central incisor. Based on clinical and radiographic examinations, diagnosis of pulp necrosis and chronic apical periodontitis was established for this tooth. As it exhibited an open apex, REP with autologous Plasma Rich in Growth Factors (PRGF) was considered as a treatment option. As the periapical lesion was so extensive, it was decided to debride it simultaneously to the REP. The patient agreed, written informed consent was obtained and the treatment was conducted in accordance with ethical principles of the University of Barcelona Dental Hospital.

On the first appointment, a pulp chamber access was created. Minimal mechanical instrumentation was performed 2 mm above the apex with an ISO #70 H-file, followed by copious irrigation with 5.25 % NaOCl solution. After drying the canal, a bi-antibiotic paste (BAP) consisting of a mixture of 250 mg of metronidazole, 250mg of ciprofloxacin, and sterile water was introduced in the canal using a #1 Buchanan hand plugger (SybronEndo, Orange, California, US) up to the apex. The access cavity was temporarily restored with 3 mm of Cavit™ (ESPE, Seefeld, Germany).

Two weeks later, during the second appointment, extraoral antisepsis was carried out using iodine solution, and local anaesthesia was administrated (articaine with epinephrine 40mg/ml + 0,01mg/ml - Artinibsa, Laboratorios Inibsa S.A., Lliçà de Vall, Barcelona, Spain). A full thickness mucoperiosteal flap was reflected from a sulcular incision extended from distal of the maxillary right canine to distal of the maxillary left central incisor. A large periapical bone defect associated with partial loss of the buccal cortical plate of the right central and lateral incisors was encountered. This fenestration was extended using a tungsten round bur 023 (Komet, Lemgo, Germany). The lesion was circumferentially separated from the bony crypt and the affected teeth using a 10 miller surgical curette (Hu-Friedy, Chicago, USA). Once removed, it was measured (11 mm x 8 mm x 12 mm), fixed in 10 % buffered formalin, and sent for histopathological examination. The surgical area was then rinsed with sterile saline solution. When the apical involvement of the lateral incisor was confirmed, a conventional endodontic treatment was performed, and its apex was resected, followed by the retrograde placement of a Mineral Trioxide Aggregate (MTA) apical barrier (Dentsply Tulsa Dental, Johnson City, TN).

With the flap raised and the apex exposed, the root canal of the central incisor was irrigated with sterile saline to remove the BAP, which was carefully collected with a surgical suction system positioned through the fenestration in the apex area. In a similar fashion, the canal was then rinsed with 5.25% NaOCl solution from coronal to apical. After complete disinfection, a final irrigation with sterile saline was performed.

Plasma rich in growth factors (PRGF®-Endoret®) was prepared according to the manufacturer’s instructions (BTI Biotechnology Institue S.L, Vitoria, Spain): Then, the plasma column was divided into two fractions: fraction 2 (F2), defined as the 2 mL of plasma just above the buffy coat, and fraction 1 (F1), defined as the plasma column above the F2. This gave a total of 8 mL of F2. The volume of F1 would depend on the haematocrit value of the patient.

**Figure 2:**
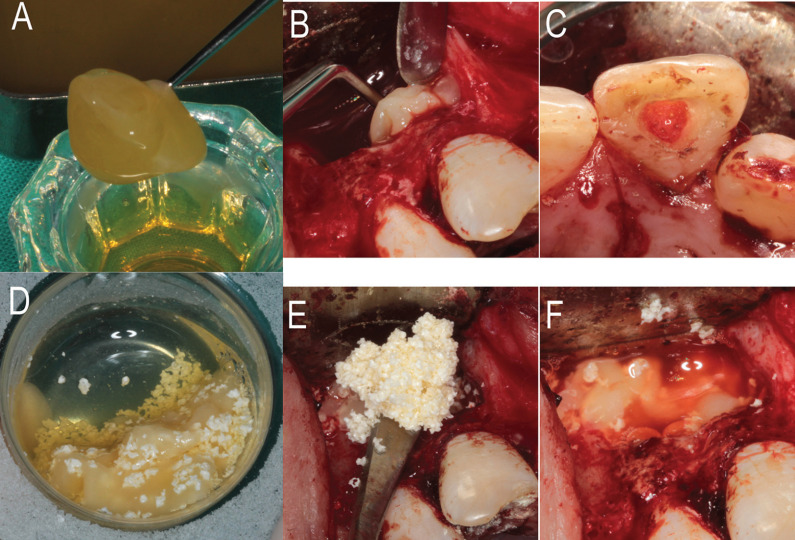
Intra-op clinical photographs of use of PRGF A) Three-dimensional clot obtained from F2 B) Filling the apical third of the root canal with the F2 clot. C) F2 clot filling the canal until 3 millimeters below the cementoenamel junction. D) Three-dimensional clot obtained by mixing F2 with (ß-TCP) E) Clot of F2 and (ß-TCP) filling intrabony defect. F) Fibrin membrane from F1 closing all defect.

The PRGF was used in the REP as follows: 2 mL of inactivated F2 was injected in the apical third of the root canal using a sterile syringe. The remaining 6 mL were gently poured in a sterile glass container and activated by adding 300 µL of 10 % calcium chloride solution, which triggered the formation of a three-dimensional fibrin clot as well as the release of growth factors and proteins from autologous platelets (Fig. 2A). This clot was divided in two: the first 3 ml clot was used to fill the apical third of the root canal from the surgical access until 3 millimetres below the cementoenamel junction (CEJ), occupying both the apical foramen and the periapical tissues (Fig. 2B,C); the other 3 ml clot was mixed with 1cc Tri-calcium phosphate (ß-TCP) (Isios, Kasios, l’Union, France), generating a new sticky bone that was used to fill the bone defect (Fig. 2D,E). The activated F1, kept at 37 ºC to obtain a haemostatic and elastic fibrin membrane, was placed over the bone graft, acting like a membrane by closing the entire defect (Fig. 2F). The mucoperiosteal flap was repositioned and held in place using 4-0 non-absorbable surgical sutures (Supramid, Aragó, Barcelona, Spain).

MTA was mixed with saline solution and placed inside of the root canal from an orthograde way, on the top of the PRGF and until 1 mm below the cemento-enamel junction (CEJ) to prevent crown staining. The F1 fibrin membrane’s consistency allowed to place the MTA safely as it permitted the total control of its final position. A moistened cotton pellet was placed in the pulp chamber, and 3 mm of Cavit™ (ESPE, Seefeld, Germany) sealed the access cavity. A postoperative radiography was taken. The patient was kept under antibiotic and analgesic coverage. Chlorhexidine gluconate solution (0.2%) was prescribed as a mouth rinse for a period of five days.

**Figure 3:**
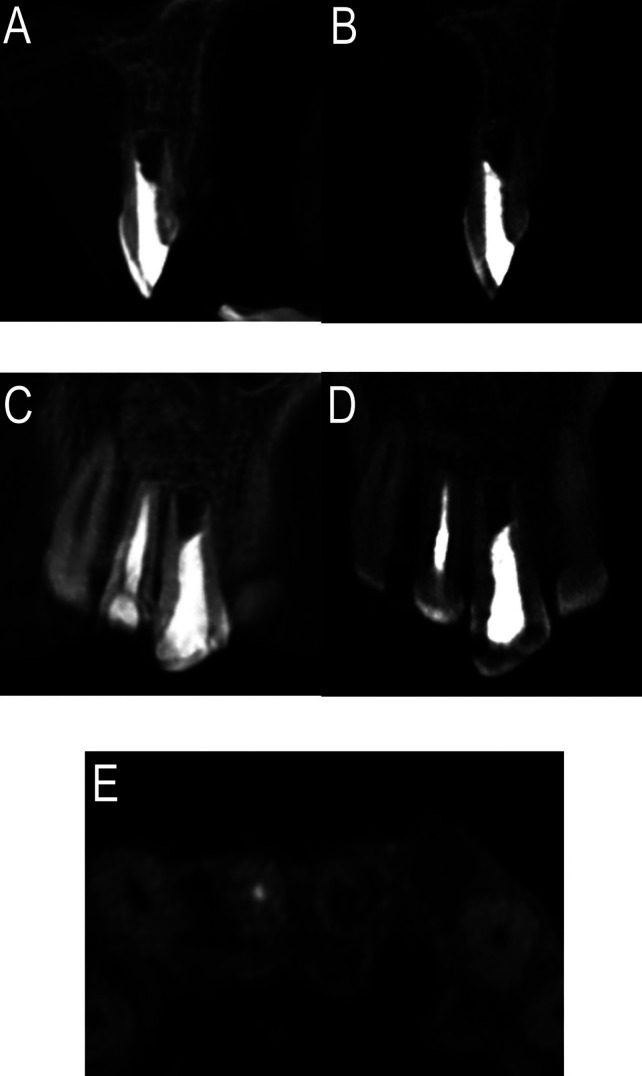
Images of cone-beam computed tomographic scan visualized by Planmeca Romexis Viewer v. 3.4.1.R. at two years (A and C) and Kodak Dental Imaging Software 3D Module v 2.4 at six years (B, D and E). A and B) Sagital slice at 1.1 level at two and six years. C and D) Coronal slice at two and six years. E) Axial slice at six years. All of them show the complete resolution of the extensive apical lesion and bone regeneration, with a slight ingrowth of bone-like tissue inside of the root canal space in the apical portion of the tooth and a slightly increase of root length at 6-years follow-up.

After a week, the suture was removed, and as the healing was uneventful, the final restoration was performed using an adhesive composite. The previously excised tissue sent for histopathological examination was diagnosed as Periapical Granuloma. In the follow-up visits at 3, 6, 12 and 24 months, there were no symptoms of pain, discomfort or inflammation. CBCT at 24 months showed the complete disappearance of the extensive apical lesion and its replacement with new bone-like tissue, thus confirming the satisfactory healing of the defect and the overall treatment successful outcome (Fig. 3A,C). CBCT at a 6-years follow-up confirmed the stability of the results and the slight ingrowth of bone-like tissue inside of the root canal space in the apical portion of the tooth, a slightly increase of root length was observed too (Fig. 3B,D,E).

## Discussion

-Root canal disinfection protocol

Historically other methods such apexification using Ca (OH)2 have been used to treat necrotic immature teeth with open apexes, but the literature shows that REP provides significantly greater tooth survival 100 % versus 77 % (3). Many of these failures are cervical fractures due to weakness caused by long-term use of calcium hydroxide (5). In the present case, the disinfection protocol of the root canal is based on a copious sodium hypochlorite irrigation combined with BAP as intracanal medicament. High (5–6%) NaOCl concentrations have been used in 36% of the published cases of REPs (6). There are reported cases of crown discoloration as a side effect after applying a triple antibiotic paste (TAP) made by ciprofloxacin, metronidazole, and minocycline, which are caused by the last one (7). This confirms the indication of using BAP instead of TAP in the REP protocol.

-PRGF use both in REP and GBR 

In the reported case, PRGF was used both as an intrapulpal and intrabony matrix as well as like a membrane to prevent epithelial migration. In REP, a matrix is necessary to provide a physiochemical and biological microenvironment that supports the growth, migration, and differentiation of stem cells (8). PRGF is versatile, easy to prepare, and can be used as a matrix both for pulp revascularization and bone regeneration. Firstly, it provides conduction: creating a three-dimensional fibrin scaffold that retains part of the released protein content, maintains the regenerative space and works as a matrix for endogenous cells. Moreover, this matrix allows the correct placement of MTA at the optimal level (8). Secondly, it provides induction, supplying growth factors and bioactive molecules which are able to influence recruitment, growth, and morphogenesis of cells in order to promote the repair of the tooth’s pulp system in REP and regeneration of bone in apical surgery (4,9).

Furthermore, in REP, platelet-rich plasma (PRP) has shown a better clinical outcome in terms of periapical healing, apical closure, and root dentinal wall thickening when compared with a blood clot and PRP is the first choice when the blood clot is insufficient, or no bleeding is found when irritating the apical tissue (10). In the present case, the size of the periapical lesion did not allow for over-extensive penetration of the canal with an endodontic file with the goal of stimulating bleeding. In this case, PRGF allowed for a sufficient and competent scaffold as well as a functional support to place the MTA.

-Guided Bone Regeneration 

Guided Bone Regeneration (GBR) techniques are needed to avoid the defect healing by fibrous connective tissue when it is too extensive (9). The combination of PRGF and Beta-tricalcium phosphate (β-TCP) as bone graft has been used in this case. Firstly, PRGF provides a provisional matrix for the attachment and migration of immune cells, fibroblasts, endothelial cells, and tissue cells. Secondly, the bone graft, which exhibits osteoconductive capability, serves as a scaffold on which cells can attach, migrate, grow, and divide. The addition of PRP to bone graft facilitates a better and faster bone regeneration compared to PRP alone (11). In addition, the use of PRGF decreases postoperative discomfort, reducing the most common inflammatory symptoms such as pain and swelling (12). When PRP is added to tricalcium phosphate 8 % to 10 %, more vital bone formation is obtained (13).

-Success of the case

In the REP literature, it has been suggested that the success in promotion of root development, thickening of the root dentinal walls, and apical closure is related to the existence of stem cells from the apical papilla (SCAP), which exist even in necrotic teeth. Their potential of self-renewal and differentiation into chondroblasts, osteoblasts, adipocytes and functional dentinogenic cells has been confirmed (14). In the present case, no mineralized tissue growth was obtained on the tooth walls or in the pulp space at two years, and a slight ingrowth of bone-like tissue from the periapical areas was observed after 6 years. As the chances of having the Hertwig’s epithelial root sheath maintained are very low, the PDL stem cells (a reservoir of multipotent postnatal stem cells present in adults (15)) could have been responsible for the healing pattern showed in terms of tissue repair. The CBCT findings (i.e., complete disappearance of the radiolucent lesion, wound healing, solving of the fenestration, and even the tooth canal’s filling with a bone-like material), combined with the absence of clinical signs and symptoms after 2 years, and maintained after 6 years, indicate the success of the procedure.

This case report proposes a single stage procedure combining REP therapy and apical surgery using PRGF as treatment of a tooth that would otherwise have had a long and complex management, with several visits separated months from one another. Within the limitations of this clinical case, a combined approach may be a good treatment modality in cases of extensive periapical lesions present in necrotic teeth with open apexes. However, further research under strict controlled conditions is needed to validate these results.
